# Synthesis of C12-C18 Fatty Acid Isobornyl Esters

**DOI:** 10.3390/molecules28227510

**Published:** 2023-11-09

**Authors:** Rongxiu Qin, Haiyan Chen, Rusi Wen, Zhongyun Liang, Zhonglei Meng

**Affiliations:** Guangxi Key Laboratory of Superior Timber Trees Resource Cultivation, Guangxi Forestry Research Institute, Nanning 530002, China; qrx20151112@126.com (R.Q.); chujin2005@163.com (H.C.); luse-rose@163.com (R.W.); liangxl1990@sina.com (Z.L.)

**Keywords:** camphene, lauric acid, titanium sulfate, fatty acid isobornyl ester, toxicity test

## Abstract

Camphene, C12-C18 fatty acids, and titanium sulfate were used as raw materials to study the synthesis of long-chain fatty acid isobornyl esters. Products were analyzed quantitatively by gas chromatography (GC), characterized by nuclear magnetic resonance spectroscopy (hydrogen and carbon), and evaluated using toxicity tests. The optimum reaction conditions were as follows: n(lauric acid):n(camphene) = 2.5:1, m(titanium sulfate):m(camphene) = 0.25:1, reaction temperature of 80 °C, and reaction time of 25 h. Under these conditions, the content of isobornyl laurate in the product was 74.49%, and the content of purified product was 95.02%. The reaction kinetics for isobornyl laurate showed an apparent first-order reaction in the first 9 h with an activation energy of 31.01 kJ/mol. The reaction conditions of myristic acid, palmitic acid, and stearic acid were similar to those of lauric acid, but the reaction time had to be increased as the molecular weight of the fatty acid increased. Toxicity tests for four types of long-chain fatty acid isobornyl esters showed that the samples had low toxicity.

## 1. Introduction

Plasticizers are polymer material-based additives that can increase the stretchability and softness of plastic products, as well as the plasticity and processability of polymer resins. In general, they are high-boiling-point and volatile liquids or low-melting-point solids.

According to their chemical structure, plasticizers can be divided into phthalic acid esters (PAEs), citric acid esters, aliphatic dibasic acid esters, or acrylates [1]. Traditional phthalate plasticizers account for ~80% of the total plasticizers produced worldwide [2]. However, traditional plasticizers have been restricted in some fields due to suspicions of carcinogenicity and toxicity.

Plasticizers prepared with renewable biomass resources (e.g., vegetable oil, lactic acid, cashew nuts, and waste edible oil) as raw materials can improve the mechanical and thermal properties of polyvinyl chloride-like traditional PAE plasticizers. However, they also have lower mobility and toxicity, so they have attracted considerable attention [3,4,5].

Short-chain fatty acids bornyl ester and isobornyl ester are widely used in chemical, pharmaceutical, fragrance, and other industries [6]. C6–C18 medium- and long-chain fatty acids terpinyl ester, fenchyl ester, bornyl ester, and isobornyl ester can be used as environmentally friendly (“green”) solvents, surfactants, and plastic agents, and have important applications in the field of medicine and high-end-material manufacturing. For example, bornyl-conjugated linoleate has an anti-tumor effect [7]. Medium- and long-chain fatty acids bornyl ester and isobornyl ester have low volatility, can be used as plastic additives to reduce the volatility and migration in plastics, and retain good plasticity.

Few studies have focused on the synthesis of borneol ester [8,9,10,11]. However, several studies have centered on the synthesis and kinetics of isoborneol acetate and other short-chain fatty acids. The research teams of Liu [12], Yuancui [6], Wenlong [13], Jinyue [14], Juntao, and others [15,16,17,18,19,20,21,22] have investigated the synthesis, separation, and purification of isobornyl acetate under different conditions. However, this process is plagued by several problems (e.g., complex preparation, high corrosiveness, and easy deactivation of the catalyst), and reports on the synthesis of medium- and long-chain fatty acid isobornyl ester are scarce.

Herein, the synthesis of isobornyl laurate using titanium sulfate as a catalyst was investigated, and high-purity isobornyl laurate products were prepared. Our study provides a theoretical basis for the development of new uses of long-chain fatty acid isobornyl esters, as well as the promotion and application of their products. Simultaneously, our research has important practical importance for the expansion of the use of green products and improving their added value.

## 2. Results and Discussion

### 2.1. Orthogonal Experiment and Reaction Kinetics

#### 2.1.1. Orthogonal Experiment

According to the orthogonal table L16 (4^4^), the reaction products were determined by gas chromatography (GC). The GC content of isobornyl laurate was calculated by the area-normalization method. The results of the orthogonal experiment are shown in Table 1.

With the GC content of isobornyl laurate as the evaluation index, the magnitude of the influence of factors examined in the orthogonal test was in the following order: molar ratio of lauric acid to camphene > reaction temperature > mass ratio of titanium sulfate to camphene > reaction time (Table 1). Among the factors, the values of A4, B4, C3, and D3 were the largest. Hence, we concluded that A4B4C3D3 was the optimal solution; that is, the molar ratio of lauric acid to camphene was 2.5, the mass ratio of titanium sulfate to camphene was 0.25, the reaction temperature was 100 °C, and the reaction time was 25 h.

According to the results of the orthogonal experiment, the dose of lauric acid was the most important factor, and increasing the molar ratio of lauric acid to camphene would increase the conversion rate of camphene. However, due to the high boiling point of lauric acid, excess lauric acid would increase the cost of recovery, so a ratio of 2.5 was deemed appropriate. Temperature was the second most important factor. Increasing the temperature would increase the reaction rate. However, too high a temperature would promote side reactions. At a reaction temperature of 80 °C, the product was light brown. With an increase in temperature, the color of the product deepened gradually. At a reaction temperature > 100 °C, the product became dark brown. The deep color increased the difficulty of decolorization and the application range of the product. Therefore, the appropriate reaction temperature was 80 °C, and the conversion rate could be increased by increasing the reaction time.

#### 2.1.2. Reaction Kinetics

**Figure 1:**
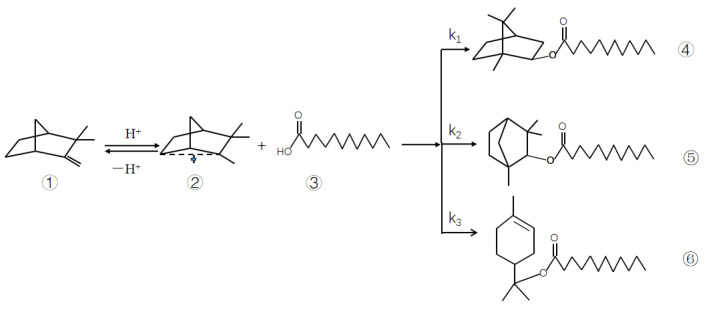
Reaction of camphene and lauric acid. Note: k_1_−k_3_ reaction rate constant; ① camphene; ② carbocation; ③ lauric acid; ④ isobornyl laurate; ⑤ fenchyl laurate; ⑥ terpinyl laurate.

Theoretically, the esterification reaction is reversible. However, if the amount of lauric acid is much greater than that of camphene, the camphene reaction is essentially complete. A small amount of camphene was present in the reactor at this time, so it can be assumed that all camphene was converted to isobornyl laurate. If the effects of mass transfer as well as internal and external diffusion are ignored, a pseudo-homogeneous model of kinetics can be established. Under optimum conditions, the conversion of camphene was 80.93%, and the selectivity of isobornyl laurate was >97.12%. Therefore, only the main chemical kinetics were studied. The reaction of camphene and lauric acid is shown in Figure 1.

The kinetics of the reaction of camphene with lauric acid can be expressed as follows:

(1) $$v_{1}=-\frac{d_{n}}{d_{t}}=kc_{1}^{\alpha }c_{2}^{\beta }$$

where *v*_1_ is the reaction rate of camphene (mol/min), *k* is the reaction rate constant, *c*_1_ and *c*_2_ represent the number of moles of camphene and lauric acid, respectively, and *α* and *β* represent the reaction order of camphene and lauric acid, respectively.

During esterification, the concentration of lauric acid is in excess. Hence, in the reaction process, the concentration of lauric acid can be considered to be a constant ($c_{2}^{\beta }$ is a constant), so Equation (1) can be simplified as follows:

(2) $$v_{1}=-\frac{d_{n}}{d_{t}}=k_{b}c_{1}^{\alpha }$$

where $k_{b}=kc_{2}^{\beta }$. If the reaction is first order (i.e., α=1), then Equation (2) can be expressed as follows:

(3) $$\frac{d_{n}}{d_{t}}=-k_{b}c_{1}$$

where the unit of $k_{b}$ is min^−1^.

**Figure 2:**
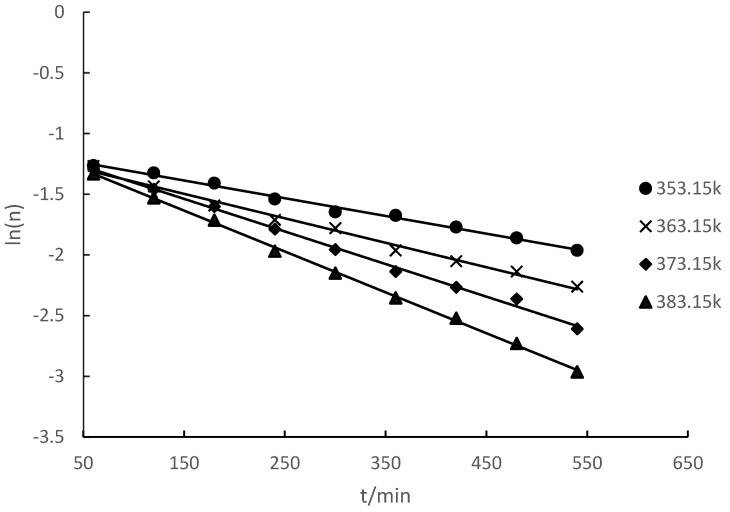
Relationship between the logarithm of the molar mass of camphene and time at different temperatures.

The esterification reaction of camphene and lauric acid was carried out at different temperatures. The amount of camphene-based substances at different reaction times was measured. The relationship between the logarithmic value ln(n) of the number of moles of camphene and time (t) was obtained (Figure 2).

Figure 2 reveals that the relationship between ln(n) and t was linear. Hence, the reaction of camphene with lauric acid to synthesize isobornyl laurate was an apparent first-order reaction. The chemical kinetics equations and rate constants for different reaction temperatures are shown in Table 2.

**Figure 3:**
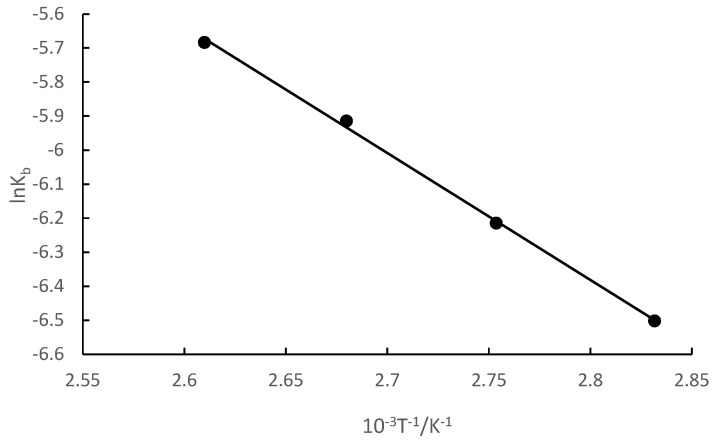
Relationship between ln*k_b_* and 1/T.

The relationship between the kinetic constant of the reaction and temperature is *k_b_* = _A_^E/RT^, where A is the pre-exponential factor. The *k_b_* value and temperature T in Table 2 were used to plot a graph of ln*k_b_* and 1/T, and the results are shown in Figure 3.

Figure 3 reveals that ln*k_b_* had a good linear relationship with 1/T. The linear equation was ln*k_b_* = −3730.3T^−1^ + 4.06, and the correlation coefficient (R^2^) was 0.9986. The activation energy (Ea) of the synthesis of isobornyl laurate could be calculated directly from the slope, and was 31.01 kJ/mol.

In conclusion, isobornyl laurate synthesized from camphene and lauric acid was an approximate first-order reaction in the early stage (9 h), but not a first-order reaction in the later stage, which may have been caused by various side reactions with an extension of reaction time. According to the amount of camphene at different temperatures, the Ea of isobornyl laurate was 31.01 kJ/mol.

### 2.2. Comparison of Test Results of Different Catalysts

We wished to investigate the effect of different catalysts on the synthesis of isobornyl laurate. Sulfuric acid was used to catalyze the synthesis of isobornyl laurate under identical conditions, and the results were compared with the blank test. The test results are shown in Table 3.

Table 3 reveals that lauric acid did not react with camphene without a catalyst. The catalytic effect of titanium sulfate was greater than that of sulfuric acid. The color of the product was darker when sulfuric acid was used as a catalyst.

### 2.3. Catalyst Life

After the reaction, the catalyst was filtered out before the product was solidified. The effect of three consecutive uses of the catalyst on the synthetic reaction was investigated. The conversion rate of camphene and the GC content of isobornyl laurate were used as assessment indices. The experimental results are shown in Table 4.

Table 4 reveals that the conversion of camphene decreased from 80.93% to 78.15% and the content of isobornyl laurate in the reaction product decreased from 74.49% to 72.35% after the catalyst had been recycled three times. These reductions were not large, and the catalyst showed a good service life.

### 2.4. Separation, Purification, and Structural Identification of Long-Chain Fatty Acid Isobornyl Ester

#### 2.4.1. Separation and Purification of Isobornyl Laurate

**Figure 4:**
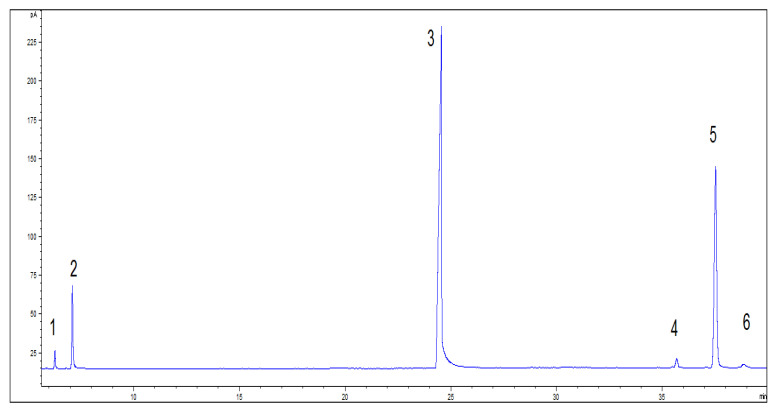
GC spectrum of camphene and lauric acid reaction products. Note: 1. tricyclene (CAS:508-32-7); 2. camphene; 3. lauric acid; 4. fenchyl laurate; 5. isobornyl laurate; 6. terpinyl laurate.

After the reaction, the catalyst was separated. The product was washed to neutral pH, and a sample was taken for GC (Figure 4). The resulting product was dissolved in a solution of ethyl acetate. This action was followed by a reaction with the fatty acids in the product by dropping in sodium hydroxide and filtering out the precipitated soap to obtain crude isobornyl laurate containing camphene, as shown in the Supplementary Materials (Figure S1). The boiling point of camphene is very different from that of isobornyl laurate, so removing camphene by fractionation is a simple process. Water vapor was passed through the crude product and camphene was brought out through the vapor (which ensured a light color of the product). After the removal of camphene, the GC content of isobornyl laurate could reach 95.02%, as shown in the Supplementary Materials (Figure S2).

#### 2.4.2. Synthesis and Separation of C14–C18 Fatty Acid Isobornyl Ester

The reaction conditions of myristic acid, palmitic acid, and stearic acid are similar to those of lauric acid. However, the reaction time must be increased because the molecular weight of the fatty acid increases. When using titanium sulfate alone as a catalyst, the molar ratio of stearic acid to camphene was 1.5, the total amount of catalyst was 25% of the mass of camphene, the temperature was 80 °C, and the reaction time was 50 h. The yield of isobornyl stearate was 67.5%, and the total yield of fenchyl stearate, isobornyl stearate, and terpinyl stearate was 75.3%, as shown in the Supplementary Materials (Figure S3). The reaction rate of long-chain fatty acids with camphene could be accelerated using a catalyst based on a titan-α-hydroxy carboxylic acid–boric acid ternary complex. For example, when this composite catalyst was composed of titanium sulfate, tartaric acid, and boric acid at a mass ratio of 1:1:0.5, the molar ratio of stearic acid: camphene was 1.5, the total amount of catalyst was 25% of the mass of camphene, the temperature was 80 °C, the reaction time was 24 h, the yield of isobornyl stearate was 67.8% and the total yield of the three esters was 72.1%.

Samples of isobornyl laurate with a GC content of 95.02% and a yield of 75.6% after the deacidification and separation of camphene were analyzed using nuclear magnetic resonance (NMR) spectroscopy. The ^1^H NMR and ^13^C NMR spectra of isobornyl laurate can be found in the Supplementary Materials (Figures S4 and S5). The NMR results were as follows: ^1^H NMR (400 MHz, CDCl_3_) δ 4.67 (dd, J = 7.9, 3.3 Hz, 1H), 2.26 (t, J = 7.5 Hz, 2H), 1.85–1.49 (m, 7H), 1.35–1.04 (m, 18H), 1.02–0.80 (m, 12H).

^13^C NMR (100 MHz, CDCl_3_) δ: 173.14, 80.56, 48.54, 46.84, 45.00, 38.81, 34.72, 33.71, 31.89, 29.58, 29.45, 29.31, 29.26, 29.14, 27.01, 25.04, 22.66, 20.07, 19.85, 14.07, 11.38.

Samples of isobornyl myristate with a GC content of 97.81% and a yield of 73.4% after the deacidification and separation of camphene were analyzed by NMR spectroscopy. The ^1^H NMR and ^13^C NMR spectra of the isobornyl myristate can be found in the Supplementary Materials (Figures S6 and S7). The NMR results were as follows: ^1^H NMR (400 MHz, CDCl_3_) δ: 4.68 (dd, J = 7.9, 3.3 Hz, 1H), 2.28 (t, J = 7.5 Hz, 2H), 1.73–1.57 (m, 6H), 1.28–1.26 (m, 22H), 1.17–0.84 (m, 15H).

^13^C NMR (100 MHz, CDCl_3_) δ: 173.39, 80.69, 48.60, 46.90, 45.04, 38.86, 34.83, 33.76, 31.93, 29.68, 29.64, 29.60, 29.47, 29.36, 29.28, 29.17, 27.04, 25.09, 22.69, 20.12, 19.89, 14.12, 11.44.

Samples of isobornyl palmitate with a GC content of 96.57% and a yield of 71.7% after the deacidification and separation of camphene were analyzed by NMR spectroscopy. The ^1^H NMR and ^13^C NMR spectra of the isobornyl palmitate can be found in the Supplementary Materials (Figures S8 and S9). The NMR results were as follows: ^1^H NMR (400 MHz, CDCl_3_) δ: 4.68 (dd, J = 8.0, 3.2 Hz, 1H), 2.27 (t, J = 7.5 Hz, 2H), 1.74–1.52 (m, 6H), 1.32–1.25 (m, 26H), 1.17–0.84 (m, 15H).

^13^C NMR (100 MHz, CDCl_3_) δ:173.39, 80.69, 48.61, 46.91, 45.05, 38.83, 34.83, 33.76, 31.93, 29.70, 29.69, 29.68, 27.04, 25.09, 22.70, 20.12, 19.89, 14.12, 11.44.

Samples of isobornyl stearate with a GC content of 95.32% and a yield of 70.5% after the deacidification and separation of camphene were analyzed by NMR spectroscopy. The ^1^H NMR and ^13^C NMR spectra of the isobornyl stearate can be found in the Supplementary Materials (Figures S10 and S11). The NMR results were as follows: ^1^H NMR (400 MHz, CDCl_3_) δ: 4.68 (dd, J = 7.9, 3.3 Hz, 1H), 2.32 (t, J = 7.5 Hz, 2H), 1.73–1.58 (m, 6H), 1.28–1.25 (m, 30H), 1.11–0.84 (m, 15H).

^13^C NMR (100 MHz, CDCl_3_) δ: 173.35, 80.67, 48.60, 46.90, 45.04, 38.86, 31.93, 29.70, 29.67, 29.64, 29.60, 29.47, 29.37, 29.28, 29.17, 27.04, 25.08, 22.69, 20.11, 19.89, 14.11, 11.43.

### 2.5. Toxicity Test

The results of the toxicity test showed that Kunming (KM) mice did not show symptoms of poisoning within 14 days of exposure. After experimental observation, the gross anatomy of surviving KM mice was examined; obvious abnormalities were not found. A test of acute oral toxicity of a sample to KM mice revealed a median lethal dose (LD50) > 5000 mg/kg·bodyweight (bw). Under the conditions of this test, the LD50 of this sample to KM mice was >500 mg/kg·bw. According to the classification standard for acute toxicity in GB/T 21605-2008 (Methods for Testing Acute Inhalation Toxicity of Chemicals) [23], the test of acute oral toxicity in this sample suggested “low” toxicity (Table 5).

## 3. Experimental Section

### 3.1. Materials and Apparatus

Commercial camphene (containing 79.8% camphene and 17.3% tricyclic terpene), lauric acid (purity ≥ 98%), myristic acid (≥98%), palmitic acid (≥98%), stearic acid (≥98%), tartaric acid (99.5%), boric acid (≥98%), titanium sulfate (≥96%), and sulfuric acid (≥98%) were obtained from Shanghai McLin Biochemical Technology (Shanghai, China). All reagents were analytically pure. Deionized water was used in all experiments.

The reaction apparatus was a chemistation (PPV-3000; Tokyo Rikakikai, Tokyo, Japan). The nuclear magnetic resonance spectrometer was an AVANCE III (300 MHz or 600 MHz) from Bruker (Billerica, MA, USA). We used a gas chromatograph (7890A series; Agilent Technologies, Santa Clara, CA, USA) equipped with quartz capillary chromatography columns (60 m × 0.25 mm × 0.25 μm) with AT-35 as the immobile phase. A gas chromatography–mass spectrometry (GC–MS) instrument (TQ456; Bruker, Billerica) equipped with BR-5 elastic quartz capillary columns (30 m × 0.25 mm × 0.25 μm) was also employed.

### 3.2. Experimental Methods

#### 3.2.1. Synthesis of Long-Chain Fatty Acid Isobornyl Ester

##### Synthesis

Camphene (10 g), long-chain fatty acids (15–37 g), and titanium sulfate (1–2.5 g) were added to a reaction bottle. The mixture underwent magnetic stirring (500 rpm) at a controlled reaction temperature (80–90 °C) and a reaction time of 15–30 h. After the reaction, the catalyst was filtered and separated. The product was transferred to a liquid-separation funnel and washed up to four times with water at 50–60 °C. The sample was dissolved in ethyl acetate for GC.

##### Separation and Purification

First, the synthetic product was dissolved in ethyl acetate. Then, sodium hydroxide solution was added. The upper layer was crude long-chain fatty acid isobornyl ester. The lower layer was long-chain fatty acid sodium solution. Finally, the crude isobornyl fatty acid was distilled in water to obtain high-purity isobornyl fatty acid.

##### Orthogonal Experiment for the Synthesis of Isobornyl Laurate

The four factors were the molar ratio of lauric acid to camphene (A), the mass ratio of titanium sulfate to camphene (B), the reaction temperature (C), and the reaction time (D). The GC content of isobornyl laurate was the evaluation index. Table 6 shows the level of orthogonal test factors.

### 3.3. Analysis and Test Methods

For GC, high-purity nitrogen was used as the carrier gas. The temperature program was an initial temperature of 70 °C (held for 2 min), with the first ramp of 5 °C/min to 150 °C (held for 3 min), followed by a second ramp of 10 °C/min to 230 °C (held for 10 min). The inlet temperature was set at 250 °C. The total flow rate was set to 130.5 mL/min, with a split ratio of 50:1 and a septum purge rate of 3 mL/min. Analytes were detected using a flame ionization detector, with a port temperature of 250 °C, hydrogen flow rate of 40 mL/min, air flow rate of 450 mL/min, and nitrogen purge rate of 25 mL/min. The injection volume was 0.2 µL.

For GC–MS, high-purity helium was used as the carrier gas. The temperature program was an initial temperature of 50 °C (held for 3 min), with a first ramp of 20 °C/min to 120 °C, followed by a second ramp of 2 °C/min to 180 °C (held for 2 min), and a third ramp of 50 °C/min to 250 °C (held for 5 min). The inlet temperature was set to 230 °C. The interface temperature was set to 250 °C.

For ^1^H NMR spectroscopy, the sample was placed in a sample tube. CDCl_3_ was added, and the sample underwent measurement with a 600 MHz NMR instrument (frequency: 600.18 MHz) in which the temperature was 296.9 K. The number of scans was 64, and the pulse width was 12.6 μs. The spectral width was 12,315.27 and the number of data points was 32,768. NMR spectra were processed by MestReNova™ (Mestrelab Research, A Coruña, Spain) and integrated after calibration, phase, and baseline calibration.

For the toxicity test, the experiment was carried out according to GB/T 21603-2008 (Test Method for Acute Oral Toxicity of Chemicals). Twenty male and female specific pathogen-free KM mice (18–22 g) were used. The sample was weighed (5.01 g), and corn oil added to prepare a sample solution of 20 mL. After thorough mixing, this solution had a final concentration of 250 mg/mL. KM mice were fed in an animal house in our laboratory for 3 days to enable them to adapt to their environment. Before the experiment, KM mice were fasted for 16 h but had free access to drinking water. The maximum dose was 5000 mg/kg·bw, and the volume was 20 mL/kg·bw by gavage. KM mice were weighed before exposure and continued to fast for 3 h after exposure. The poisoning and death of KM mice were recorded. The observation period was 14 days. After the observation period, the surviving KM mice were killed, and gross anatomy was documented. If abnormal tissues or organs were found, histopathology was undertaken.

## 4. Conclusions

Our study draws three main conclusions. First, the optimum reaction conditions for the synthesis of isobornyl laurate were as follows: n(lauric acid):n(camphene) = 2.5:1, m(titanium sulfate):m(camphene) = 0.25:1, reaction temperature = 80 °C, and reaction time = 25 h. Under these conditions, the GC content of isobornyl laurate was 74.49% and was 95.02% after purification. The purified product was identified as isobornyl laurate by 1H NMR and 13C NMR spectroscopy. Analysis of kinetics revealed an apparent first-order reaction (9 h) with an Ea of 31.01 kJ/mol.

Second, the reaction conditions of myristic acid, palmitic acid, and stearic acid were similar to those of lauric acid, but the reaction time needed to increase with an increase in molecular weight of the fatty acid. The reaction rate of myristic acid, palmitic acid, and stearic acid could be improved by using a composite catalyst containing titanium sulfate, tartaric acid, and boric acid.

Third, toxicity tests using four types of fatty acid isobornyl esters revealed no toxicity symptoms for KM mice within 14 days of exposure. According to the classification standard for acute toxicity in GB/T 21605-2008 (Methods for Testing Acute Inhalation Toxicity of Chemicals), the test of acute oral toxicity of this sample suggested “low” toxicity.

## Figures and Tables

**Table 1:** Orthogonal experiment.

Number	Camphene (g)	Molar Ratio of Lauric Acid to Camphene (A)	Mass Ratio of Titanium Sulfate to Camphene (B)	Temperature (°C) (C)	Time (h) (D)	Camphene Conversion (%)	GC Content of Isobornyl Laurate (%)
1	10	1	0.1	80	15	27.52	23.75
2	10	2	0.2	80	25	66.36	62.58
3	10	2.5	0.25	80	30	85.78	79.72
4	10	1.5	0.15	80	20	56.48	51.62
5	10	1.5	0.25	100	15	71.29	64.72
6	10	2.5	0.20	90	15	52.43	47.36
7	10	2	0.15	110	15	70.95	66.58
8	10	1	0.25	110	25	76.95	67.16
9	10	2.5	0.10	110	20	69.75	66.47
10	10	1	0.20	100	20	80.86	74.53
11	10	1.5	0.20	110	30	60.92	54.81
12	10	1.5	0.10	90	25	66.61	61.75
13	10	2	0.10	100	30	68.49	65.06
14	10	2	0.25	90	20	69.95	66.59
15	10	2.5	0.15	100	25	78.49	71.59
16	10	1	0.15	90	30	38.65	31.91
	K1	197.35	217.03	222.19	202.41		
	K2	232.9	221.97	207.61	259.21		
	K3	260.81	239.28	276.17	263.35		
	K4	269.93	282.71	255.02	236.02		
	R	72.58	65.68	68.56	60.94		
	Order	1	3	2	4		
	Scheme	4	4	3	3		

**Table 2:** Reaction kinetics equation and rate constant at different temperatures.

Temperature/k	Rate Constant/*k_b_* Min^−1^	Kinetic Equation
353.15	0.0015	lnn = −0.0015t − 1.1672, R^2^ = 0.99
363.15	0.002	lnn = −0.002t − 1.1949, R^2^ = 0.99
373.15	0.0027	lnn = −0.0027t − 1.1216, R^2^ = 0.99
383.15	0.0034	lnn = −0.0034t − 1.1691, R^2^ = 0.99

**Table 3:** Comparison of experimental results for different catalysts.

Number	Catalyst	Camphene (g)	Molar Ratio of Lauric Acid: Camphene	Mass Ratio of Catalyst: Camphene	Temperature (°C)	Time/(h)	GC Content of Isobornyl Laurate (%)
1	No catalyst	10	2.5	0	80	25	-
2	titanium sulfate	10	2.5	0.10	80	25	55.49
3	sulfuric acid	10	2.5	0.10	80	25	48.31

**Table 4:** Reuse test of catalysts.

Number	Camphene Conversion (%)	GC Content of Isobornyl Laurate (%)
1	80.93	74.49
2	79.47	73.58
3	78.15	72.35

**Table 5:** Acute oral toxicity test of four fatty acids of isobornyl ester at a dose of 5000 mg/kg·bw.

Name	Sex of KM Mice	Number of Animals	Number of Dead KM Mice	Percent Mortality
Isobornyl laurate	Female	10	0	0
	Male	10	0	0
Isobornyl myristate	Female	10	0	0
	Male	10	0	0
Isobornyl palmitate	Female	10	0	0
	Male	10	0	0
Isobornyl stearate	Female	10	0	0
	Male	10	0	0

**Table 6:** Factors and levels used in the orthogonal array design.

Level	Factor A	Factor B	Factor C	Factor D
1	1:1	0.1:1	80	15
2	1.5:1	0.15:1	90	20
3	2:1	0.20:1	100	25
4	2.5:1	0.25:1	110	30

## Data Availability

The data presented in this study are available from the corresponding author upon reasonable request.

## Supplementary Materials

The following supporting information can be downloaded at: https://www.mdpi.com/article/10.3390/molecules28227510/s1, Figure S1. GC spectrum of products after removal of lauric acid. Figure S2. GC spectrum of products after removal of lauric acid and camphene. Figure S3. GC spectrum of product after removal of stearic acid. Figure S4. 1H NMR spectrum of isobornyl laurate. FigureS5. 13C NMR spectrum of isobornyl laurate. Figure S6. 1H NMR spectrum of isobornyl myristate. Figure S7. 13C NMR spectrum of isobornyl myristate. Figure S8. 1H NMR spectrum of isobornyl palmitate. Figure S9. 13C NMR spectrum of isobornyl palmitate. Figure S10. 1H NMR spectrum of isobornyl stearate. Figure S11. 13C NMR spectrum of isobornyl stearate.
