# Supplementary material for: Synthesis of C12-C18 Fatty Acid Isobornyl Esters
Source: Molecules. 2023 Nov 9;28(22):7510. doi: 10.3390/molecules28227510 (PMC10673531; doi:10.3390/molecules28227510)
Supplement: Supplementary file 1 [file molecules-28-07510-s001.zip › molecules-2688360-supplementary.pdf]

Supporting Information

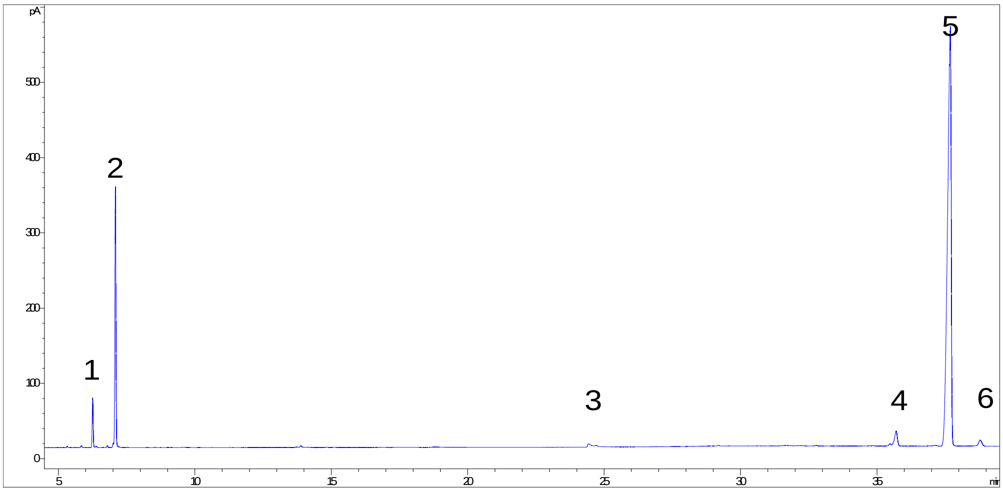

Figure S1. GC spectrum of products after removal of lauric acid.

Note: 1. tricyclene(CAS:508-32-7); 2. camphene; 3. lauric acid; 4. Fenchyl laurate; 5. isobornyl laurate; 6. terpinyl laurate.

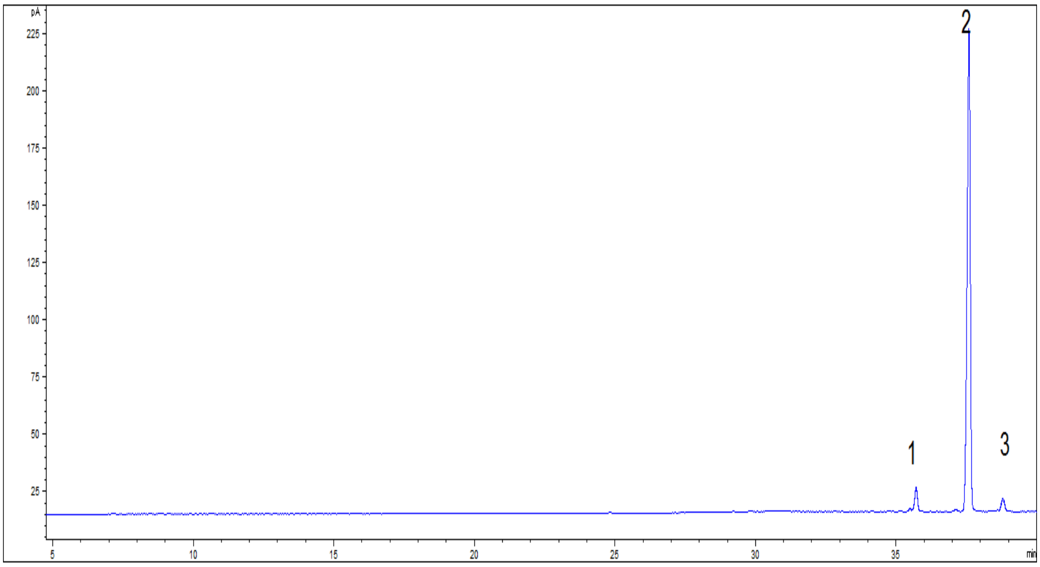

Figure S2. GC spectrum of products after removal of lauric acid and camphene.

Note: 1. Fenchyl laurate; 2. isobornyl laurate; 3. terpinyl laurate.

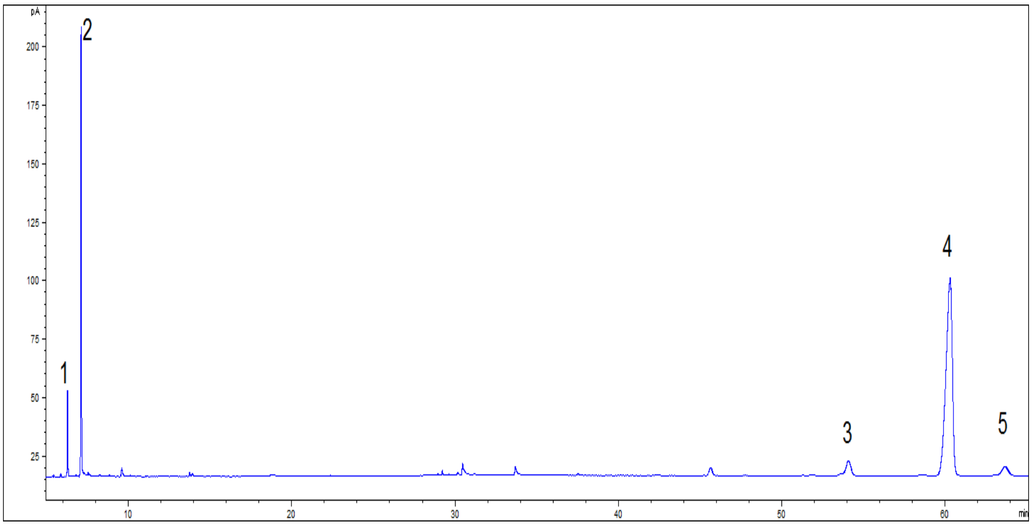

Figure S3. GC spectrum of product after removal of stearic acid. Note: 1. Tricyclene(CAS:508-32-7); 2. Camphene; 3. fenvalerate stearate; 4. isobornyl stearate; 5. terpinyl stearate.

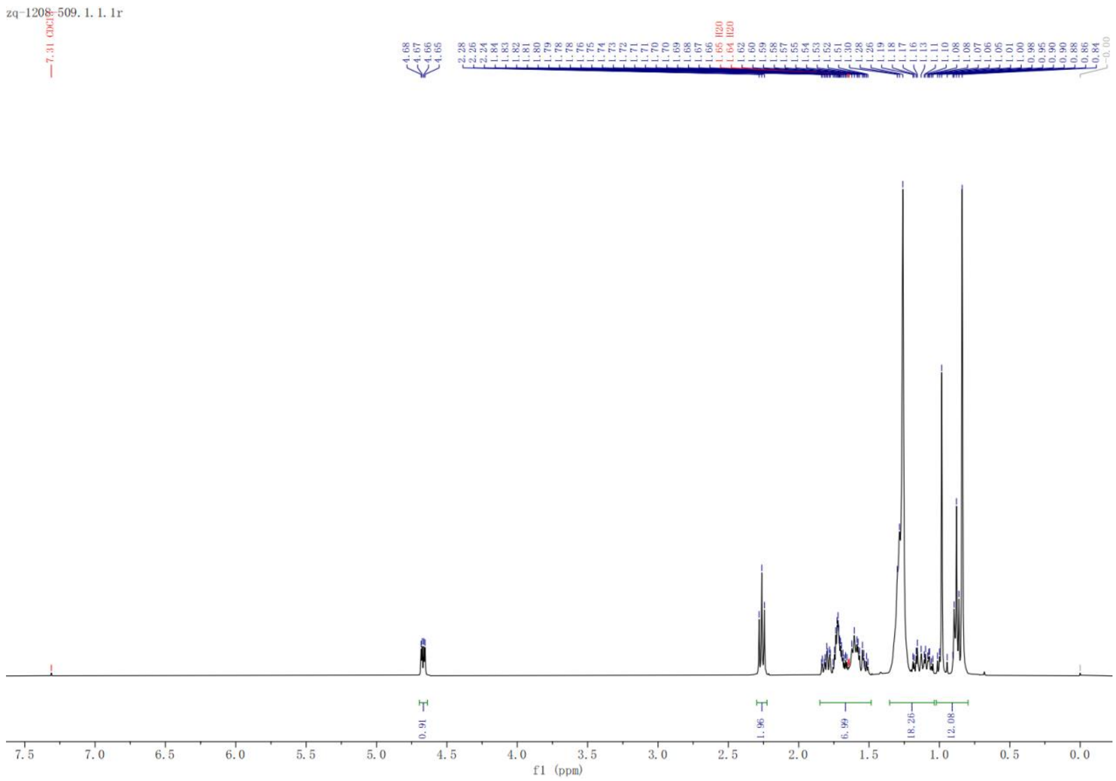

Figure S4. ^{1}H NMR spectrum of isobornyl laurate.

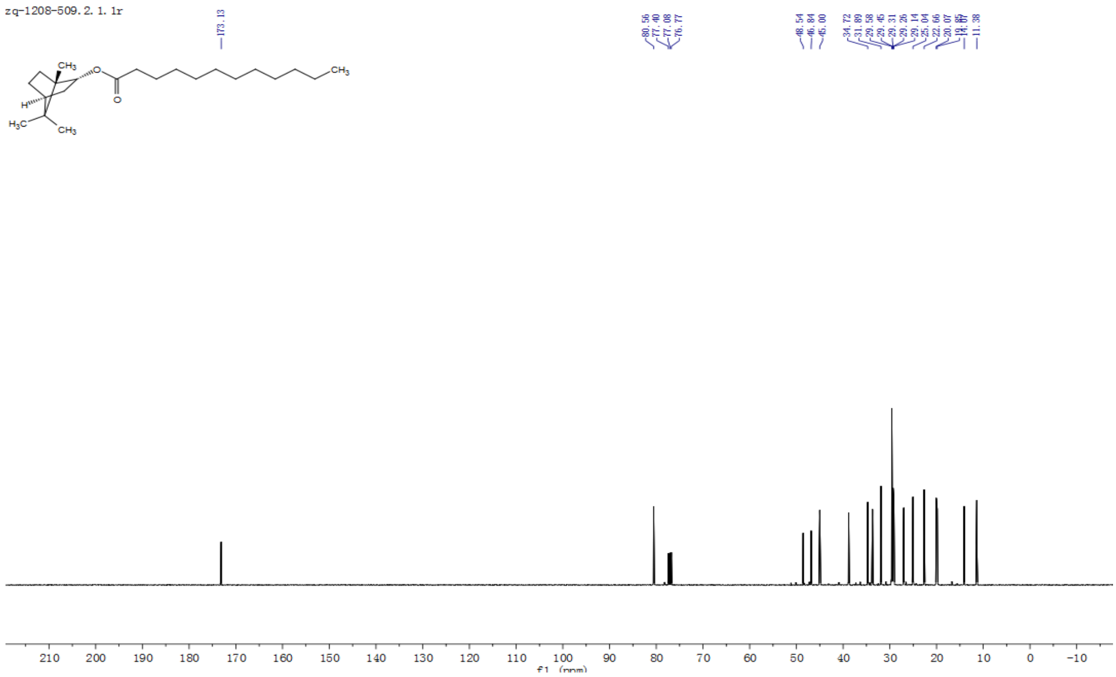

Figure S5. ^{13}C NMR spectrum of isobornyl laurate.

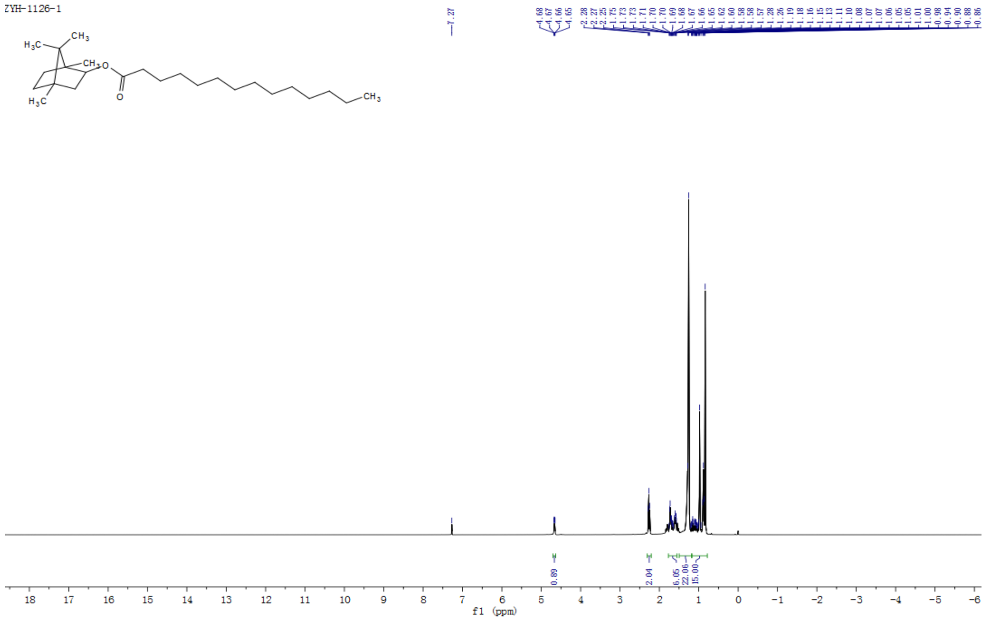

Figure S6. ^{1}H NMR spectrum of isobornyl myristate.

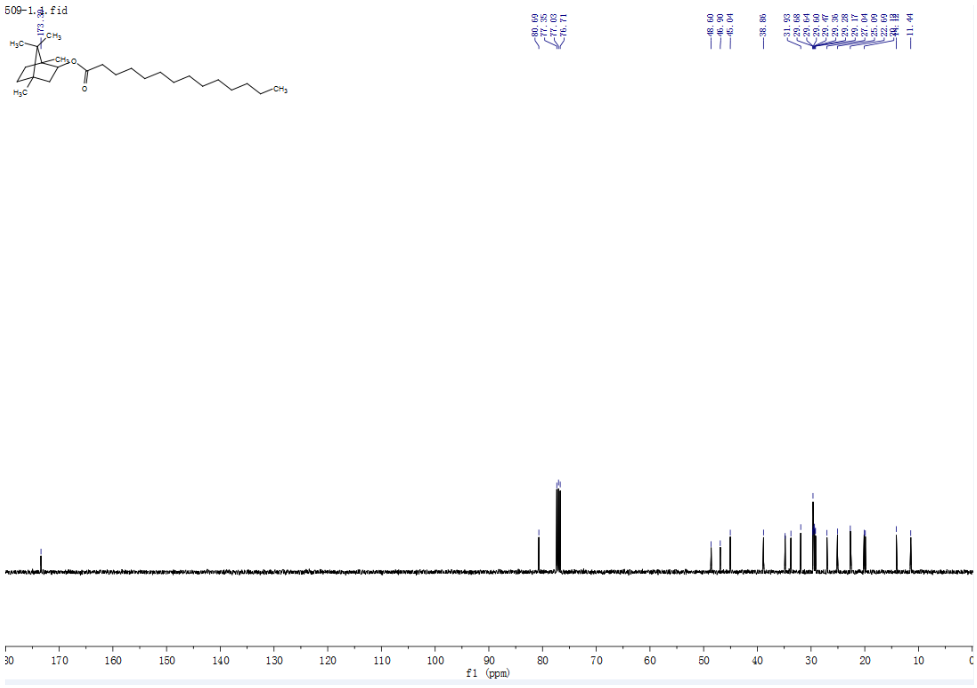

Figure S7. ^{13}C NMR spectrum of isobornyl myristate.

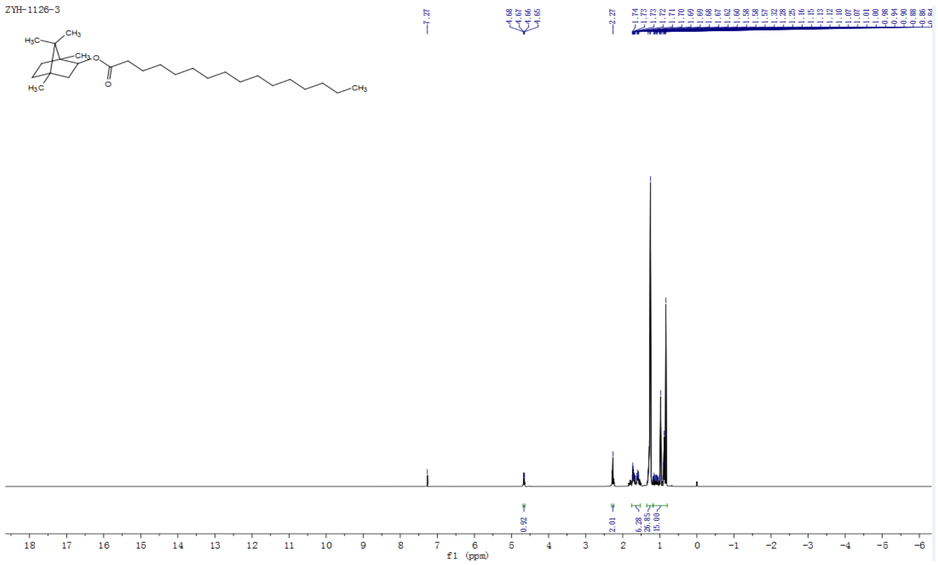

Figure S8. ^{1}H NMR spectrum of isobornyl palmitate.

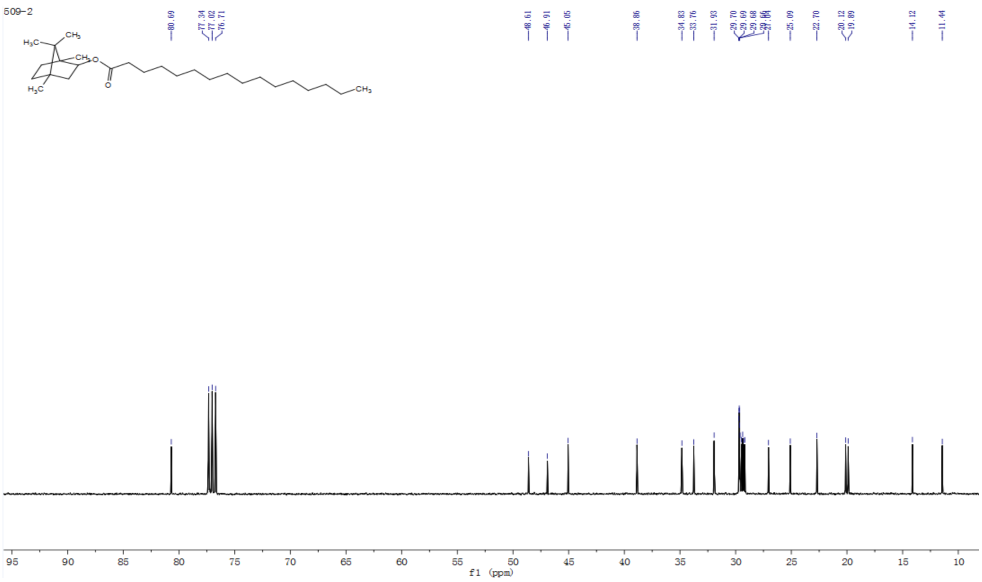

Figure S9. ^{13}C NMR spectrum of isobornyl palmitate.

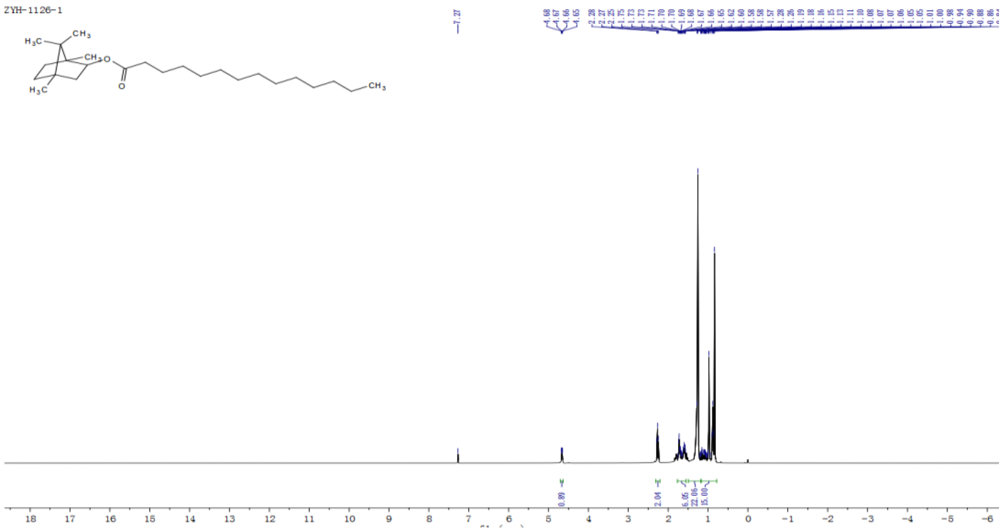

Figure S10. ^{1}H NMR spectrum of isobornyl stearate.

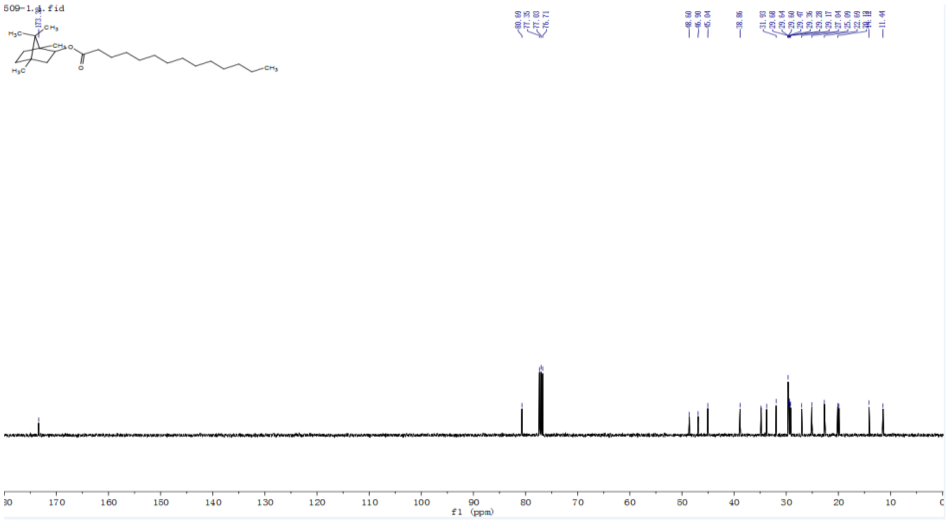

Figure S11. ^{13}C NMR spectrum of isobornyl stearate.
